# Multicentre consensus recommendations for skin care in inherited epidermolysis bullosa

**DOI:** 10.1186/1750-1172-9-76

**Published:** 2014-05-20

**Authors:** May El Hachem, Giovanna Zambruno, Eva Bourdon-Lanoy, Annalisa Ciasulli, Christiane Buisson, Smail Hadj-Rabia, Andrea Diociaiuti, Carolina F Gouveia, Angela Hernández-Martín, Raul de Lucas Laguna, Mateja Dolenc-Voljč, Gianluca Tadini, Guglielmo Salvatori, Cristiana De Ranieri, Stephanie Leclerc-Mercier, Christine Bodemer

**Affiliations:** 1 Dermatology Unit, Bambino Gesù Children's Hospital, IRCCS, Piazza S. Onofrio, 4, 00165 Rome, Italy; 2 Laboratory of Molecular and Cell Biology, Istituto Dermopatico dell’Immacolata, IDI-IRCCS, Rome, Italy; 3 Department of Dermatology, Necker–Enfants Malades Hospital, National reference centre for Genodermatoses (MAGEC), 149 rue de Sèvres, 75015 Paris, France; 4 Université Paris Descartes - Sorbonne Paris Cité, Institut Imagine, Paris, France; 5 Dermatology University Clinic, Hospital de Santa Maria, Centro Hospitalar Lisboa Norte EPE, Lisbon, Portugal; 6 Department of Dermatology, Hospital Infantil del Niño Jesús, Madrid, Spain; 7 Department of Dermatology, Hospital Infantil La Paz, Madrid, Spain; 8 Department of Dermatovenereology, University Medical Centre Ljubljana, Ljubljana, Slovenia; 9 Section of Dermatology, Fondazione IRCCS Cà Granda-Ospedale Maggiore Policlinico di Milano, Milan, Italy; 10 Neonatal Intensive Care Unit, Department of Medical and Surgical Neonatology, Bambino Gesù Children’s Hospital, IRCCS, Rome, Italy; 11 Clinical Psychology Unit, Bambino Gesù Children’s Hospital, IRCCS, Rome, Italy

**Keywords:** Inherited epidermolysis bullosa, Multidisciplinary management recommendations, Skin care, Wound care, Itch, Pain, Therapeutic education, Disease burden, Quality of life, Continuity of care

## Abstract

**Background:**

Inherited epidermolysis bullosa (EB) comprises a highly heterogeneous group of rare diseases characterized by fragility and blistering of skin and mucous membranes. Clinical features combined with immunofluorescence antigen mapping and/or electron microscopy examination of a skin biopsy allow to define the EB type and subtype. Molecular diagnosis is nowadays feasible in all EB subtypes and required for prenatal diagnosis. The extent of skin and mucosal lesions varies greatly depending on EB subtype and patient age. In the more severe EB subtypes lifelong generalized blistering, chronic ulcerations and scarring sequelae lead to multiorgan involvement, major morbidity and life-threatening complications. In the absence of a cure, patient management remains based on preventive measures, together with symptomatic treatment of cutaneous and extracutaneous manifestations and complications. The rarity and complexity of EB challenge its appropriate care. Thus, the aim of the present study has been to generate multicentre, multidisciplinary recommendations on global skin care addressed to physicians, nurses and other health professionals dealing with EB, both in centres of expertise and primary care setting.

**Methods:**

Almost no controlled trials for EB treatment have been performed to date. For this reason, recommendations were prepared by a multidisciplinary team of experts from different European EB centres based on available literature and expert opinion. They have been subsequently revised by a panel of external experts, using an online-modified Delphi method to generate consensus.

**Results:**

Recommendations are reported according to the age of the patients. The major topics treated comprise the multidisciplinary approach to EB patients, global skin care including wound care, management of itching and pain, and early diagnosis of squamous cell carcinoma. Aspects of therapeutic patient education, care of disease burden and continuity of care are also developed.

**Conclusion:**

The recommendations are expected to be useful for daily global care of EB patients, in particular in the community setting. An optimal management of patients is also a prerequisite to allow them to benefit from the specific molecular and cell-based treatments currently under development.

## Background

Inherited epidermolysis bullosa (EB) refers to a clinically and genetically heterogeneous group of rare disorders characterized by fragility of the skin and mucous membranes. Based on the site of blister formation, four major types of EB are currently distinguished: EB simplex (EBS), junctional EB (JEB), dystrophic EB (DEB), and Kindler syndrome (KS); each one is then subdivided into several subtypes based on the mode of transmission and a combination of phenotypic, immunofluorescence, ultrastructural and molecular findings [1]. Once the cleavage level and protein expression pattern has been determined, molecular testing is the most accurate diagnostic procedure for EB subtype definition. Furthermore, it is required for DNA-based prenatal diagnosis. The extent and severity of skin and mucous membrane lesions and multiorgan involvement vary markedly in the different EB types and subtypes and in relation to age [1-5]. In several EB subtypes, the cutaneous and extracutaneous manifestations and complications lead to a significant morbidity and even to premature death. The rarity of EB and the phenotypic variability challenge the appropriate care of these patients.

Despite the preclinical development of different molecular and cell-based treatment strategies, no cure is still available for EB [6]. In the absence of a specific therapy, patient management is currently centered on preventive and skin care measures, and early recognition and symptomatic treatment of complications. As almost no controlled trials for EB treatment have been performed to date [7], measures for EB management mainly rely on the experience of physicians, nurses and other involved healthcare professionals. The development of consensus recommendations based on literature data and shared expertise among experts from different countries represents an important step towards improved standard of care and quality of life for affected patients and their caregivers. Consensus recommendations based on best evidence available have been recently generated by an international panel of EB experts [8]. They comprise 17 items centered on wound care and related general health and patient issues. In addition, comprehensive best practice guidelines addressing all aspects of oral health care in EB have been published [9]. Finally, guidelines for wound and skin care in the major EB types have been generated by clinical experts and made available on the Debra International website [10]. These documents provide an invaluable support for the treatment of specific and major manifestations of EB disease. However, they do not address several equally important disease-related aspects such as the multidisciplinary management organization, therapeutic patient education, care of disease burden and continuity of care. We have therefore developed a list of consensus recommendations on current best practices for global skin care in EB. They are conceived to provide a practical support for day-to-day management of patients both in a hospital setting and in community care. Our aim has been to consider all aspects of patient life affected by the skin disease and also the family burden. The recommendations are addressed to all healthcare professionals dealing with EB both in centres of expertise and in community care: physicians (dermatologists, neonatologists, pediatricians, internists, pain relief doctors, anesthetists, surgeons, general practitioners), nurses, dieticians, psychologists, physical and occupational therapists, and social workers. They are expected to be useful also for patients and their caregivers.

## Methods

A multidisciplinary group of experts in EB diagnosis and care from different European countries (France, Italy and Portugal) met in Paris in October 2011 at the occasion of the closing meeting of the EU-supported project “Together Against Genodermatoses”. It was decided to generate a list of consensus recommendations on current best practices for global skin care in patients affected with all types of EB. A list of topics was identified and subgroups of experts revised the pertinent literature (English, French and Italian) since January 2000. Older articles deemed relevant by the experts were also considered. Literature search included all published recommendations and guidelines on EB care. A provisional list of recommendations for each topic was then prepared. The resulting global document was revised by additional experts from Spain and Slovenia and then circulated among a second group of experts using an online-modified Delphi method to generate consensus. The final document is reported below.

### Multidisciplinary management of EB patients

The fragility of the skin and mucous membranes in EB patients results in the involvement of many organs and systems [1-5,11-18] (Tables 1 and 2). In addition, disease manifestations vary upon the age of the patients and the EB type and subtype.

**Table 1:** Major epidermolysis bullosa complications affecting the skin, eye and ENT area

**Tissue/organ/system**	**Major complications**	EB type/subtype*
Skin	Fluid loss	Lethal acantholytic EB, JEB-H, EBS-PA, JEB-PA
	Chronic/infected wounds	RDEB-SG, RDEB-I, RDEB-O, JEB-H, JEB-nH, EBS-AR, DEB-Pr, DDEB-G, EBS-DM
	Exuberant granulation tissue	LOC, JEB-H, JEB-nH
	Atrophic scars, post-inflammatory pigmentary changes	DEB, JEB, EBS
	Poikiloderma/diffuse skin atrophy	KS
	Excessive/hypertrophic scarring	RDEB-SG, RDEB-I, DEB-Pt, DEB-Pr, RDEB-O
	Albopapuloid lesions	DEB
	Milia	DEB, JEB, EBS, KS
	Palmoplantar keratoderma	EBS, JEB-nH, KS
	Aplasia cutis congenita	EBS-PA, JEB-PA, DEB, other JEB and EBS subtypes
	EB nevi	JEB, DEB, EBS
	Basal cell carcinomas	EBS-DM
	Squamous cell carcinomas	RDEB-SG, RDEB-O, KS, JEB-nH, RDEB-I
Skin adnexa	Onychodystrophy, nail shedding or loss	DEB, JEB, EBS-MD, EBS-DM, EBS-PD, EBS-AR, EBS-O, KS
	Scarring alopecia	JEB-nH, RDEB-SG, JEB-H, JEB-PA, RDEB-O
	Alopecia universalis	Lethal acantholytic EB
	Hypotrichosis	EBS-PD
Oral cavity	Microstomia, ankyloglossia, obliteration of the oral vestibules	RDEB-SG, RDEB-I, RDEB-O
	Enamel hypoplasia	JEB, EBS-MD
	Multiple caries and tooth decay	DEB, JEB
	Periodontitis	KS
External eye	Corneal erosions	RDEB-SG, JEB-H, RDEB-O, RDEB-I, JEB-nH, EBS-DM, KS
	Blepharitis, corneal scarring and/or pannus formation	RDEB-SG, RDEB-I, JEB-H, RDEB-O, JEB-nH
	Symblepharon	LOC, RDEB-SG, RDEB-I, JEB-H, JEB-nH
	Ectropion/exposure keratitis	JEB-H, RDEB-SG, KS
	Diminished vision/blindness	RDEB-SG
	Conjunctival granulation tissue	LOC
External ear	External auditory canal narrowing/conductive hearing loss	RDEB-I
Nose	Nare narrowing (granulation tissue)	JEB-H, JEB-nH, LOC

*The epidermolysis bullosa (EB) types and subtypes which present a higher frequency of a given complication are listed first.

EBS, epidermolysis bullosa simplex; EBS-PA, EBS with pyloric atresia; EBS-DM, EBS, Dowling-Meara; EBS-MD, EBS with muscular dystrophy; EBS-AR, EBS, autosomal recessive; EBS-PD, plakophilin deficiency; EBS-O, EBS, generalized other; JEB, junctional epidermolysis bullosa; JEB-H, JEB, Herlitz; JEB-nH, JEB, non-Herlitz; JEB-PA, JEB with pyloric atresia; LOC, laryngo-onycho-cutaneous syndrome; DEB, dystrophic epidermolysis bullosa; DDEB, dominant DEB; DDEB-G, DDEB, generalized; RDEB-SG, recessive DEB, severe generalized; RDEB-O, recessive DEB, generalized other; RDEB-I, recessive DEB, inversa; DEB-Pr, DEB, pruriginosa; DEB-Pt, pretibial DEB; KS, Kindler syndrome.

**Table 2:** Other major extracutaneous complications of epidermolysis bullosa

**Tissue/organ/system**	**Major complications**	EB type/subtype*
Gastrointestinal tract	Pyloric atresia	JEB-PA, EBS-PA
	Esophageal stenosis/strictures/web formation	RDEB-SG, RDEB-I, KS, RDEB-O
	Chronic constipation/fecal impaction	RDEB-SG, RDEB-I, RDEB-O, DDEB, JEB-H, JEB-nH, EBS-DM, EBS-MD
	Gastroesophageal reflux disease	RDEB, JEB-nH, EBS-DM, EBS-MD, JEB-PA, JEB-H, DDEB
	Anal fissures/stenosis	RDEB-SG, RDEB-I, RBED-O, KS
	Protein-loosing enteropathy	JEB-PA, EBS-PA, JEB-H, JEB-nH
	Colitis/diarrhea	KS, RDEB, JEB-PA
Genitourinary tract	Urethral strictures, meatal stenosis	JEB-H, RDEB-SG, JEB-PA, JEB-nH, LOC, KS
	Genitourinary malformations, ureteral/ureterovesical junction obstruction/stenosis, recurrent cystitis	JEB-PA, EBS-PA
	Vulvar/vaginal scarring/strictures	RDEB-I, KS
	Renal failure	RDEB-SG, JEB-PA, JEB-nH
Upper respiratory tract	Tracheolaryngeal stenosis/acute respiratory failure	JEB-H, LOC, EBS-MD, lethal acantholytic EB, EBS-DM
Musculoskeletal system	Osteopenia and osteoporosis	RDEB-SG, RDEB-O, JEB-nH
	Limb flexion contractures	RDEB-SG
	Digit contractures/pseudosyndactyly	RDEB-SG, RDEB-O, RDEB-I, KS
	Mitten deformities	RDEB-SG
	Muscular dystrophy	EBS-MD, EBS-PA
Hematopoietic system	Multifactorial anemia	RDEB-SG, JEB-H, JEB-PA, EBS-PA, JEB-nH, EBS-AR, EBS-DM, RDEB-O
Heart	Dilated cardiomyopathy	RDEB-SG, JEB-nH, EBS-MD
Endocrine	Delayed puberty, amenorrhea	RDEB-SG, RDEB-O
Systemic complications	Sepsis	JEB-H, JEB-nH, RDEB-SG, EBS-DM
	Failure to thrive, growth retardation	JEB-H, JEB-PA, EBS-PA, RDEB-SG, JEB-nH, RDEB-O, EBS-AR, EBS-DM, RDEB-I

*The EB types and subtypes which present a higher frequency of a given complication are listed first.

EBS, epidermolysis bullosa simplex; EBS-PA, EBS with pyloric atresia; EBS-DM, EBS, Dowling-Meara; EBS-MD, EBS with muscular dystrophy; EBS-AR, EBS, autosomal recessive; EBS-PD, plakophilin deficiency; EBS-O, EBS, generalized other; JEB, junctional epidermolysis bullosa; JEB-H, JEB, Herlitz; JEB-nH, JEB, non-Herlitz; JEB-PA, JEB with pyloric atresia; LOC, laryngo-onycho-cutaneous syndrome; DEB, dystrophic epidermolysis bullosa; DDEB, dominant DEB; DDEB-G, DDEB, generalized; RDEB,recessive DEB; RDEB-SG, recessive DEB, severe generalized; RDEB-O, recessive DEB, generalized other; RDEB-I, recessive DEB, inversa; DEB-Pr, DEB, pruriginosa; DEB-Pt, pretibial DEB; KS, Kindler syndrome.

### General principles

• Treatment of EB patients should be performed in centres of expertise adhering to the recommendations for quality criteria issued by the European Union Committee of Experts on Rare Diseases (EUCERD) [19].

• Centres of expertise should also guarantee the continuity of care between pediatric age and adulthood [20].

• A coordinated multidisciplinary approach must be adopted (Table 3).

**Table 3:** Specialists involved in multidisciplinary epidermolysis bullosa care

**Physicians**	**Other professionals**
Dermatologist	Specialist nurse
Neonatologist/pediatrician/internist	Dietitian
Pathologist	Psychologist
Medical geneticist	Dental hygienist
Otolaryngologist	Physical therapist
Ophthalmologist	Occupational therapist
(Pediatric) surgeon	Speech therapist
Orthopedic surgeon	Social worker
Plastic surgeon	
(Pediatric) gastroenterologist	
Dentist	
(Pediatric) anesthetist	
Endocrinologist	
Neurologist	
Radiologist	
Pain relief doctor	
Cardiologist	
Nephrologist	
Oncologist	

• The coordinator of the team should be a dermatologist in order to ensure both an integrated management and continuity of care with the community healthcare system [21-30].

• The multidisciplinary management is centered on the patient; therefore, it is a tailored treatment for each patient.

• The multidisciplinary team should be specifically trained and regularly updated [19].

## Care of the EB newborn and infant

Several procedures performed in newborns and infants can severely injury the EB fragile skin and require specific and adapted care measures [10,31,32]. In addition, the skin immaturity in pre-term newborns affected with EB and the ongoing functional skin adaptation to the extra-uterine environment in term babies demand particular caution in skin handling and care. Skin and mucosal lesions predispose to recurrent and potentially life-threatening infections and cause loss of fluids and electrolytes which can lead to dehydration and electrolyte imbalance. Although the extent and severity of skin and mucous membrane involvement is extremely variable, general and specific measures for skin care of the newborn and infant affected with EB can be recommended.

### General principles

• The first multidisciplinary care should be provided independently from the diagnostic definition of the EB type and subtype.

• Diagnosis should be performed as soon as possible in order to implement the most appropriate treatment, communicate the diagnosis and specific information to the parents. Methods and criteria for diagnosis have been regularly updated by an International Consensus Conference [1].

• The communication of the diagnosis should involve the dermatologist and the neonatologist and should be addressed to both parents. The information should be delivered gradually and adapted to the family socio-cultural level.

• A psychologist should support the family (see paragraph “Care of disease burden”).

• Caregiver education should start promptly (see paragraph “Therapeutic patient education”).

• Members of the multidisciplinary team usually involved in EB care in infancy are neonatologist/pediatrician, dermatologist, anesthetist, pathologist, medical geneticist, psychologist and specialized nurses.

### General measures

• Blood sampling for complete blood count, electrolytes, C-reactive protein, urea, creatinine, total serum protein and albumin, iron, zinc, and, whenever required, blood cultures. Swabs for culture should be taken from infected wounds.

• In severely affected newborns, a venous access should be guaranteed through placement of an umbilical venous catheter. Whenever required, this will be followed by elective insertion of an indwelling central venous catheter (tunnelled external design such as Broviac catheters).

• The following measures should be adopted to prevent blistering:

✓ the baby should not be placed systematically in an incubator unless needed for reasons such as prematurity [10]: heat and humidity can lower the threshold for blistering. An overhead heater can be used, cautiously regulated.

✓ Naso- and oro-pharyngeal suction should be avoided. If required, a soft catheter is chosen, and minimal suction pressure exerted [31].

✓ The umbilical cord should be secured with a ligature, avoiding the use of plastic clamps which rub the skin [10,31].

✓ The use of clips should be avoided, and name band put on clothing instead of wrist.

✓ Electrodes should be of small size; the adhesive rim should be removed allowing only the lubricated central portion to be in contact with the skin and the electrode should then be secured with a non-adhesive dressing (e.g. Mepilex®, Mölnlycke) [33].

✓ Clip sensors should be used for pulse oximetry.

✓ For blood pressure monitoring, thick padding is recommended before applying the blood pressure cuff.

✓ When possible, skin-to-skin contact (kangaroo care) with parents should be encouraged.

✓ There is no contraindication to immunization for infectious diseases.

### Skin care

**Figure 1:**
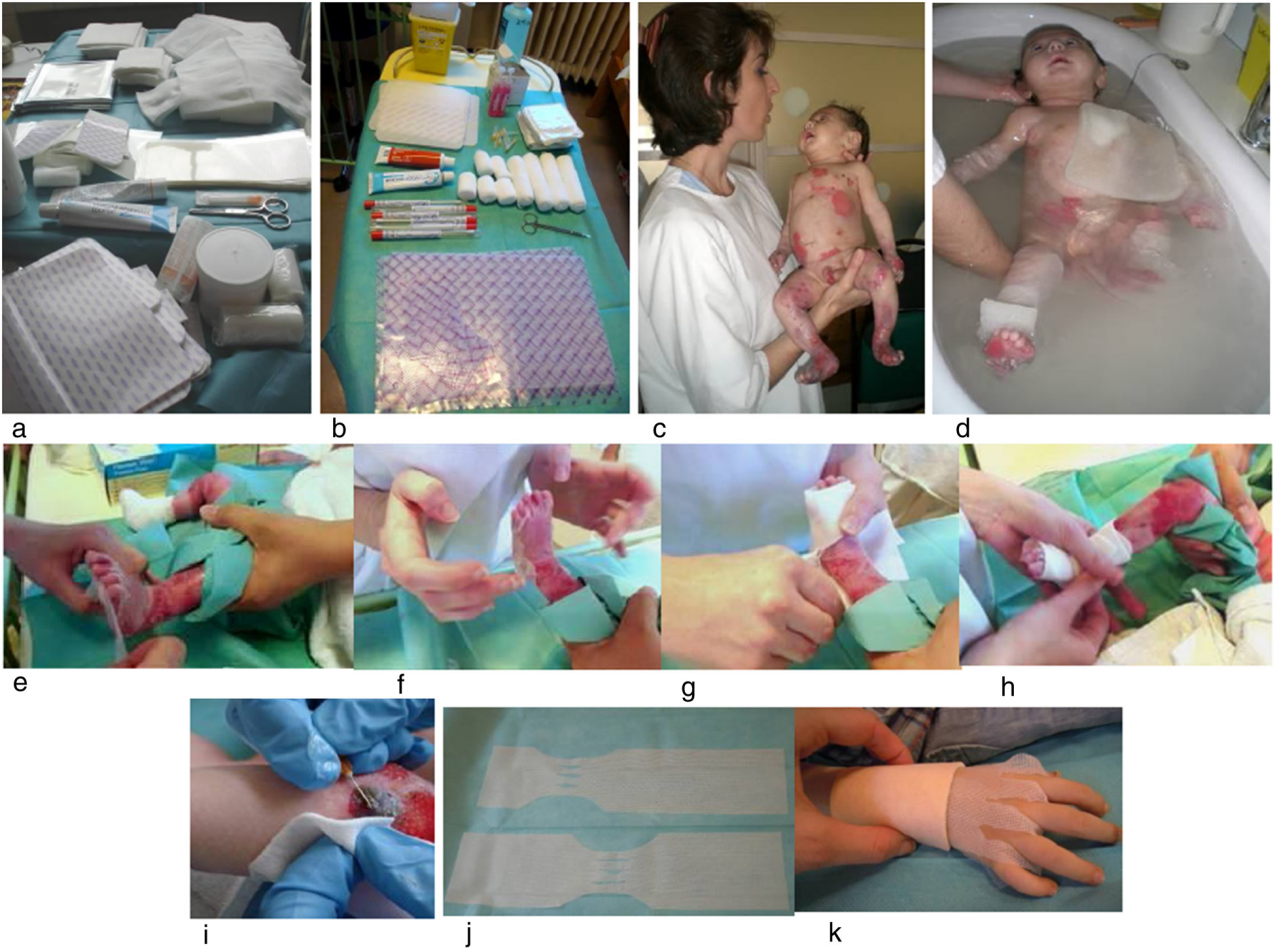
**Inherited epidermolysis bullosa: wound care. (a, b)** Dressing cart prepared in advance for patient dressing: soft silicone foams (*), petroleum jelly (<), emollient cream (>), antimicrobial cream (◊), gauzes, tubular bandages, needles and swabs for culture (∇). **(c)** How to hold the baby: one hand behind the head and the other one behind the buttocks. **(d)** Bathing the baby also facilitates atraumatic removal of dressings which float into the water. **(e-h)** Wound care with non-adherent soft silicone dressings and thin polyurethane-soft silicone foams. **(i)** Lancing and draining of a haemorrhagic blister. **(j-k)** Soft silicone foams specifically modelled for hand dressing **(j)**, and hand dressing to separate fingers and prevent early digit fusion **(k)**.

• For clothing, a front-fastening babygro is easier to put on and remove. It should be turned inside out to prevent the seams from rubbing skin [31]. If available, DermaSilk® (Alpretec) underclothes and gloves can be used as they combine silk properties with the protective activity of an antimicrobial agent, in addition to being seam-free. Alternatively, Tubifast (Mölnlycke) garments and gloves can be used [10].

• Disposable nappies can be used, they should first be lined with a soft material (e.g. soft silicone contact layer or foam such as Mepitac®, Mölnlycke) in order to reduce skin rubbing from the elastic edges. Nappies with Velcro fasteners prove safer as it is less likely for the securing tapes to adhere to the skin [10,31].

• Particular attention should be paid to avoid friction when handling the baby: he/she should be nursed on neonatal incubator mattress, and lifted by sliding hands below the mattress or using the sheet [10]. To handle the naked infant, the nurse/caregiver should roll the baby away, place one hand behind his/her neck and head and the other one behind his/her buttocks, let the baby roll back and then lift him/her [31,32] (Figure 1).

• Adhesive tapes must be avoided. Soft silicone fixation tapes providing atraumatic removal (e.g. Mepitac®) are recommended to secure devices such as electrodes, catheters, tubes, and probes. For removal they should be rolled out gently rather than lifted.

• Silicone Medical Adhesive Removers (SMARs), such as Appeel® (CliniMed) or Niltac®, should be used to remove electrodes or accidentally applied tapes or dressing/clothing adhered to wounds. If SMARs are not available, liquid and white soft paraffin in equal parts facilitate atraumatic removal [10,34,35].•Regular bathing in tepid to slightly warm water is recommended (Figure 1), the frequency will be adapted to each case. If the infant presents crusted lesions, an emollient/oil-based cleanser should be used, while infected wounds should be treated with an antiseptic (e.g. containing 0.1% chlorhexidine). After bathing, the skin should be dried by gentle padding with a towel.

• Diaper area cleansing should be performed with liquid and white soft paraffin in equal parts or with an emollient/oil-based cleanser [10,31].

• Principles for blister, aplasia cutis congenita and wound treatment are similar to those applying to children and adults (see paragraph “Wound care”).

• Aplasia cutis congenita and blistering involving hands or feet or digit degloving following delivery require specific dressing in order to prevent early digit fusion [10]. The separation should be performed by using easily modelled dressings, such as soft silicone foams (e.g. Mepilex® or Mepilex Lite®), which are cut into strips (Figure 1). If advanced dressings are not available, paraffin-impregnated gauzes can be used, and should also be cut into thin strips. Attention should be paid to keep the first finger extended and separated from the rest of the hand.

• The diaper area is constantly subject to handling, exposed to urine, feces and to the diaper occlusion effect. We prefer to treat the less exuding erosions with paraffin-impregnated gauzes, replaced at each nappy change. Alternatively, a soft silicone primary wound dressing (e.g. Mepitel®) or hydrogel-impregnated gauzes (e.g. Intrasite® Conformable, Smith & Nephew) can be used. In more exuding lesions, an antiseptic and silicone foams (e.g. Mepilex® or Mepilex®Transfer) are indicated.

### Feeding modalities

• In less severely affected newborns breast-feeding is possible: soft paraffin can be applied on the nipple and breast as well as on the infant face and lips to reduce friction from rooting reflex [31]. The mother should be trained to handle the baby and breast feed him. When breast-feeding proves traumatic, oral feeding remains the best option, also allowing to add supplements in malnourished infants.

• Commercially available teats should be softened with warm boiled water. The teat hole can be enlarged or extra holes may be created to facilitate sucking. Lips can be protected with petroleum jelly to avoid the skin sticking to the teat [31].

• A Haberman feeder reduces the sucking effort and its long teat avoids nose trauma from the bottle collar [10,31].

• A few infants may require naso-gastric feeding. A small soft polyurethane tube fixed for few weeks will minimize internal damage and reduce the trauma [31].

• For nutritional principles see paragraph “Nutritional aspects”.

### Follow-up

• The infant should be discharged home when the general health condition is stable and the parents are adequately educated and confident to care for their baby. This decision is taken jointly by the dermatologist and neonatologist/pediatrician. The involvement of social workers and psychologists in the organization of the discharge is helpful.

• A first follow-up visit with a specialized team (usually: dermatologist, pediatrician, EB nurse, psychologist) will be organized in one or two weeks for severe EB subtypes (e.g. Dowling-Meara EBS, JEB and recessive DEB). It should comprise a complete clinical examination (skin/mucosae, nutrition, pain, etc.), dressing, and evaluate and pursue the therapeutic education of the parents. If the infant care is correctly performed the next appointment should be in one month, then every three months during infancy. In mild EB subtypes the follow-up visits will be every 3–6 months.

### EB care from childhood to adult

Additional problems in skin and wound management during childhood to adulthood include, in particular in Dowling-Meara EBS, generalized forms of JEB and recessive DEB (RDEB) [4,5,12,13,17,36-38]:

• reduced patient compliance to care and claim for self-care;

• chronic pain;

• presence of chronic wounds and their susceptibility to infection;

• risk of cancer development;

• chronic itching;

• anemia and malnutrition secondary to oral and gastrointestinal involvement and chronic wounds, in turn negatively interfering with wound healing;

• psychological problems related to both disease acceptance and social relationship (school, hobbies, job, etc.).

#### General principles

• The members of multidisciplinary team vary upon the EB type and patient and family needs. In mild EBS subtypes (e.g. localized and generalized non-Dowling-Meara EBS), the dermatologist ensures the follow-up with the help of a specialized nurse. In severe EBS forms (such as Dowling-Meara EBS or EBS with muscular dystrophy), JEB and generalized DEB subtypes the core members are the dermatologist and the pediatrician supported by the EB nurse and dietician. Other specialists (ophthalmologist, dentist, digestive surgeon, psychologist, physiotherapists, etc.) are involved depending on disease complications.

• A regular follow-up is required to evaluate skin and mucosal conditions, general health status, and specific problems encountered by the patient and his/her caregivers. Follow-up planning should be performed taking into account the EB type, disease complications, family and patient compliance and specific complains. After childhood, EBS and mild DEB patients are generally seen by an EB specialist once a year; JEB and generalized RDEB should be seen at least twice a year. More frequent follow-up visits (e.g. every month) are required for the most severe patients in order to fasten wound healing and to early diagnose and treat squamous cell carcinoma.

#### General measures

• Monitoring of severe EBS variants (e.g. Dowling-Meara EBS or EBS with muscular dystrophy), JEB, and generalized RDEB subtypes comprises at follow-up visits: a complete blood count, electrolytes, total serum protein and albumin, iron, iron-binding capacity, ferritin, erythrocyte sedimentation rate, C-reactive protein, liver function tests, urea, creatinine and, if required, zinc, selenium, folate and vitamins (A, B_6_, C, D and E) [22,39]. Swabs for culture should be taken from infected and critically colonized wounds, as blood cultures in case of sepsis suspicion.

• Patient adherence to therapy should be regularly checked. Caregiver and patient compliance and experience should be always taken into account in designing the care plan.

• Immunization schedule for infectious diseases should be regularly continued. Chicken pox vaccination is recommended.

• Preventive measures to reduce the onset of new lesions are summarized in Table 4[10,31,32,40].

**Table 4:** Preventive measures to reduce the onset of new lesions during daily life

**Direct skin protection**	• Protect vulnerable skin sites, (e.g. knees and elbows) with soft silicon contact layers, silicon sheets or strips (e.g. KerraPro®) or thick padding
	• Use gloves when the child begins to crawl/walk and lifelong during sports or hobbies (e.g. DermaSilk® or Tubifast®)
	• Avoid hard shoes with internal seams, tight clothes and clothes with raised seams, tags in contact with the skin
	• Use protective padding for shoes, such as a poron insole or orthotic device
	• Pad the frame at the nose bridge and over the ears of eye glasses
**Other measures**	• Ensure that toys are frequently cleaned and in soft material without traumatic angles
	• Prefer hobbies and sports at low risk of skin trauma (e.g. adapted gym, swimming, ping-pong, reading, singing, playing music, electronic toys and informatics)
	• House air conditioning and other cooling measures in hot climates

#### Skin care

• Regular patient follow-up by the dermatologist comprises the assessment of the entire body including the scalp, external ear, genital/anal area, oral cavity and nostrils.

• A dermatological management is necessary in all types of EB and skin lesions.

• An appropriate analgesia should be performed before any procedure, such as bathing or dressing (see paragraph “Pain management”).

• For the hygiene, a mild antiseptic cleanser (e.g. chlorhexidine 0.1% or polyhexanide, sodium hypochlorite 5–10 ml in 5 L of water, acetic acid ≤0.25%) should be used for extended and/or critically colonized/infected lesions [39,41]. An emollient/oil-based cleanser should be chosen for xerotic skin and hyperkeratotic or crusted lesions.

• Bathing frequency depends on the type of dressing and lesion characteristics: in case of infected wounds or dressings which stick to the lesions (e.g. paraffin-impregnated gauzes) bathing should be performed every other day; when advanced dressings are employed bathing can be delayed until once a week.

• New blisters should be lanced (finger prick lancet, sterile large-bore needle or scalpel blade) (Figure 1) and drained. The blister roof should be left in place to facilitate re-epithelialization, to reduce infection risk and pain [10,31,32,42].

• Daily use of emollients and moisturizing products is strongly recommended for xerotic skin in order to reduce blistering, pain and itching [31].

• Regular photoprotection is mandatory in patients affected by KS who present photosensitivity.

• Courses of topical keratolytic agents (e.g. urea, salicylic acid, ammonium lactate) are helpful to treat palmoplantar hyperkeratosis and/or keratoderma [40]. Their concentration depends on the patient age, rhythm of application, treatment duration and local tolerance. Particular caution is required for young children.

• Hyperhidrosis, frequent in EBS patients, should be treated in order to reduce/prevent blistering and itching. Corn flour is an inexpensive and easily available remedy [10,40], other absorbent powders (e.g. Zeasorb®, Stiefel Laboratories) may also be useful. An additional option is iontophoresis [43]. Furthermore, the use of botulinum toxin may be considered for severe plantar hyperhidrosis [43,44]. Silver-lined or Dermasilk socks keep feet dry and comfy, provide anti-friction action and reduce the risk of bacterial overgrowth [10,40].

• EB nevi should undergo a regular clinical and dermoscopic follow-up [11]. Although a slightly increased risk of melanoma has been reported only for RDEB, atypical pigmented lesions undergoing significant morphological changes should be biopsied [45] and references herein].

• No specific treatment is required for milia.

• Mucosal care:

• Oral management should adhere to the recently published best practice guidelines for oral health care in EB [9].

• Conjunctiva should be regularly lubricated, in particular in JEB or RDEB patients, with lanolin and preservative-free eye drops or gels, e.g. containing hyaluronic acid, polyethylene/propylene glycol or carbomer [45].

• Nasal lubricants are more frequently indicated for JEB or RDEB patients; regular ointment containing vitamin E or simply petroleum jelly can be helpful.

### Wound care

The choice of dressings varies upon the type and site of the lesions, but also product availability [8,10,42,46,47]. Advanced dressings delay the frequency of dressing change, thus reducing pain and manipulation-related risk of blistering and infections. In addition, non-adherent dressings proved superior in reducing pain at dressing removal. However, systematic literature reviews showed only a modest advantage for advanced dressings (e.g. hydrogels, hydrofibers and foams) compared to paraffin-impregnated gauzes in accelerating healing of non-EB chronic wounds (venous leg ulcers, pressure sores, etc.) [48]. As to topical antibiotics and antiseptics, their role in healing of leg ulcers remains to be proved [49]; there is a good evidence of their usefulness for the treatment of superficial skin infections [50]. The debridement of the wounds is important to accelerate the healing process and to prevent infection: some dressings such as hydrogels, polymeric membranes and hydrofibers are helpful [8,10,46,47]. In case of multiple and deep necrotic lesions, mechanical debridement should be performed gently in the theatre with analgesia.

#### Dry to lightly exuding wounds

• Non adhesive soft silicone or lipido-colloid contact layers [e.g. Mepitel®, Adaptic® touch (Systagenix), Urgotul® (Urgo Medical), Silflex® (Advancis Medical)], thin polyurethane-soft silicone foams (e.g. Mepilex® Lite), and hydrogels (e.g. Intrasite® Conformable) appear to be the most suitable in these lesions [8,10,46,51]. Hydrogel dressings should be changed daily or as soon as they become dry. The other types could be changed every 3–4 days.

• The dressing choice depends on the affected site: e.g. flexible dressings should be used mainly for the folds. Soft bordered materials [e.g. Mepilex® Border, Alleyvn® Gentle Border (Smith & Nephew)] easily adapt to the different skin sites but they may be too sticky and thus require a primary contact layer in order to protect fragile skin [10].

#### Heavy exuding wounds

• Heavy exuding wounds present a high risk of infection and require specific dressings. Either hydrofiber dressings (e.g. Aquacel®, Convatec) or soft silicone foam with super-absorbers (e.g. Cutimed® Siltec, BSNmedical), able to absorb the abundant exudates, should be preferred. Soft silicone foams [e.g. Mepilex®, Mepilex® Transfer, and Advazorb Silflo® (Advancis Medical)] and polymeric membranes (PolyMem®, Ferris Mfg. Corp.) are also indicated [8,10,46,51]. The soft silicone foams are also suited for digits and folds. On the other hand, PolyMem® is not easily retained on the wounds and requires a secondary dressing to hold in place. Also Mepilex® Transfer needs a secondary dressing to absorb exudates.

#### Critically colonized and infected wounds

• To early diagnose a critically colonized or infected wound the following parameters and features should be considered:

✓ wound history: several week duration, recent size extension and exudate increase;

✓ wound bed: presence of debris, dead slough, friable tissue and bad smelling;

✓ wound margins and surrounding skin: oedema, erythema, higher temperature than the healthy skin.

• In the presence of the above mentioned criteria, swabs for culture should be taken after washing the lesion with normal saline and then the treatment should be started based on clinical features [41].

• Wounds should be cleaned with mild antiseptics, such as chlorhexidine 0.1% or polyhexanide, sodium hypochlorite at a concentration of 5–10 ml in 5 liters of water, or acetic acid at ≤0.25% for 15–20 minutes/day [39,41].

• In case of lesions at risk of infection, the use of lipid-stabilized hydrogen peroxide cream (Crystacide®) has been proposed [41]. An aqueous solution of eosin (2%) is employed in some countries with the aim to reduce the exudate, keeping in mind that it has no antiseptic properties. In other countries, medical-grade honey in ointments (e.g. Mesitran S®, Medloc) or dressings (e.g. Algivon®, Advancis Medical; Medihoney®, Derma Sciences) are available and used with the aim to reduce the risk of infection and promote wound debridement [10,41,52,53].

• The use of silver-containing creams (e.g. silver sulfadiazine) or dressings (e.g. Mepilex®AG; Urgotul®silver/SSD; PolyMem®silver, Aquacel®Ag) has been advocated [10,41]. However, there is no clear evidence that silver-containing products can prevent wound infection or improve healing rates of leg ulcers [52] and references herein]. Importantly, silver plasma level should be checked in case of large surface and/or prolonged treatment because of the risk of silver absorption and related toxicity [54,55]. In children, the use of silver-containing products should be very limited in time and treated surface.

• The dressings for infected wounds are the same used for heavy exuding wounds, but they should be changed daily.

• Principles for the use of topical and systemic antibiotics/antimicrobials are summarized in Table 5[8,41,56].

**Table 5:** Principles for use of antibiotics/antimicrobials in wound treatment

**Topical agents**	• Restrict the use to critically colonized and infected wounds
	• Prefer agents which do not have a systemic formulation (e.g. fusidic acid, mupirocin)
	• Use for short periods and rotate to avoid resistances and sensitizations
	• Consider retapamulin as a second line treatment for resistant Gram positive bacteria
**Systemic agents**	• Administer in multiple infected lesions
	• Start early in malnourished and/or non compliant patients and in infants
	• Prescribe antibiotics according to the result of culture
	• Prefer narrow spectrum antibiotics

#### Hyperkeratotic and crusted lesions

• Warty and crusted lesions require an accurate treatment and follow-up because they are itchy and can mask an underlying squamous cell carcinoma. The crusts and hyperkeratosis should be regularly removed. Frequent application of emollient creams and bathing twice a week are indicated to this purpose.

#### Exuberant granulation tissue

• Short courses of very potent topical glucocorticoid ointments are reported as effective in reducing the exuberant granulation tissue frequently observed in JEB [8,10].

#### General remarks

• The dressings of all types of wounds should be checked daily and the change frequency modified based on wetting and smelling.

• Paraffin-impregnated gauzes (e.g. Jelonet®, Smith & Nephew) or medicated gauzes (e.g. Fitostimoline® containing an aqueous extract of Triticum vulgaris, or Connectivine®, Fidia, containing hyaluronic acid) can be used when advanced dressings are not available [8,10]. They require a secondary dressing and should be changed on a daily basis, increasing wound manipulation, pain and indirectly also the risk of infection. These products adhere to the wound bed. In order to reduce pain and trauma, dressing removal requires prolonged soaking with distilled water or saline solution or bathing [8,10].

• In RDEB patients, finger and toe dressing should be regularly performed as described for the neonatal period, to delay digit fusion and pseudosyndactyly [10].

• Tubular bandages of various sizes and heights according to the affected body area (e.g. Elastomul®, Tubifast® or Self-fix®) should be used for dressing retention to prevent slipping and further trauma. Tight bandages must be avoided as they can induce blistering by rubbing the skin [8,10]. If available, Dermasilk® or other specifically designed garments, without silver, can also be useful to retain the dressings in place.

### Itch management

Itching is common in EB patients with dry/atrophic skin, multiple warty lesions, during late phase of wound healing, or following sensitization to topical treatment. Pruritus may also develop in the absence of an identifiable etiology [8,10]. Itching is frequently chronic, severe and unresponsive to conventional treatments. Furthermore, a rare subtype of DEB, DEB pruriginosa, is characterized by onset of severe and unremitting pruritus from infancy to adulthood [57,58]. Itch-induced scratching damages the skin, thus increasing blistering and susceptibility to infection. General measures and therapeutic options to manage itch are summarized in Table 6[8,10,59-64].

**Table 6:** Chronic itch management

**General measures**	• Bathing in tepid water with syndet/oil cleanser and skin hydration with emollients
	• Overheating and dry environment avoidance
	• Relaxation techniques and patient education to cope with the vicious itch–scratch cycle
**Therapeutic options**	• Short courses of topical mid-potency steroids
	• Sedating antihistamines (e.g. hydroxyzine) and/or tricyclics with anti-H1 antihistaminic action (doxepin) as first-line treatment*
	• Low-dose gabapentin (Neurontin®) or pregabalin (Lyrica®) as second-line treatment
	• Anti-inflammatory agents (e.g. cyclosporine, thalidomide or topical tacrolimus) to be cautiously considered as third-line treatment only in severe cases**

*Data from randomized trials are lacking to support the efficacy of antihistamines in pruritic conditions other than urticaria.

**Caution should be paid in using immunosuppressive drugs because of the carcinogenesis risk.

### Management for surgical procedures

#### General principles

• Surgery in general anaesthesia should be limited to strictly necessary procedures and organized jointly by the surgical team (surgeon, anaesthesiologist and nurse) and the EB team coordinator.

• Whenever necessary and feasible, the different surgical treatments should be performed at one time in order to reduce the risks linked to anaesthesia.

• A tailored management should be planned for each patient after a careful evaluation.

#### General measures

• When a surgical procedure in general anesthesia is planned, a multidisciplinary re-assessment should be performed a week or two ahead of the surgery date to verify the general health conditions of the patient [33].

• Blood sampling should be performed before surgery to evaluate and treat anemia.

• Before the procedure, the surgical team, including the anaesthesiologist and the case manager, should clearly explain the possible problems and complications linked to the procedure, paying particular attention to those related to EB disease.

#### Specific measures

• Anxiolytic administration can be helpful in the pre-operative stage.

• The measures to be adopted in the operating theatre are summarized in Table 7[33-66].

**Table 7:** Patient management in the operating theater

**Procedure**	**Measures to be adopted**
**Operating table**	• Place an anti-decubitus mattress and cushion on the table
	• Use the sheet to lift the infant and move him/her to the operating table; older patients should move themselves
	• Pad trauma-exposed sites (e.g. chin, occiput, elbows, heel, hands, feet)
**Premedication**	• Administer oral premedication 45 minutes prior surgery in order to reduce/prevent:
	✓ Patient anxiety (midazolam 0.5 mg/kg)
	✓ Oral secretion (atropine 40 mcg/kg)
	✓ Gastro-esophageal reflux (ranitidine 1 mg/kg)
	✓ Vomiting (metoclopramide 150 mcg/kg)
	• Prefer intravenous induction in presence of intravenous line, otherwise inhalational anaesthesia. In the latter case, protect the face from the mask with silicon foam (e.g. Mepilex®) or a water-based lubricant.
	• Protect the eyes with a moisturizing ophthalmologic gel and the eyelids with moistened gauzes
**Patient monitoring**	• Use tape with a silicon contact layer (Mepitac®) to fixe all tubes (e.g. endotracheal tube) and catheters
	• Lubricate all tubes with a water-based lubricant
	• Remove the adhesive part of electrodes allowing only the lubricated central portion to be in contact with the skin; then secure with a non adhesive dressing (e.g. Mepilex ®)
	• Use clip sensors for pulse oximetry
	• Use a lubricated disposable thermometer
	• Pad the skin with cotton or advanced dressings under the blood pressure cuff
	• Use bipolar diathermy to avoid a monopolar pad
	• Avoid carefully all kinds of trauma and friction for the entire duration of surgery
**Intubation**	• Evaluate microstomia, esophageal strictures and prominent incisors in RDEB* patients who need intubation
	• Prefer fiberoptic-assisted intubation to laryngoscopy in case of difficult intubation
**Recovery room**	• Administer a moderate sedation before emergence to avoid cutaneous lesions due to irritability
	• Perform tracheal aspiration gently using soft and small tubes

*RDEB, recessive dystrophic epidermolysis bullosa.

• After surgery, the intravenous line should be kept in place as long as possible to be used for transfusion, perfusion or other systemic therapy (iron, albumin, antibiotics).

• Fiberoptic intubation appears to be a good technique to minimise frictional trauma and to reduce the risk of blistering, whilst safely securing the difficult airway [67].

### Pain management

Pain is constant in EB patients since birth, and its management is a major therapeutic focus conditioning the daily care. A tailored approach should be planned for each patient considering the different types of pain, and the treatment efficacy should be evaluated regularly.

#### General principles

• Pain is acute, chronic and related to procedures (e.g. bathing, dressing, surgical procedures, etc.). Psychological pain and anxiety contribute constantly to worsen organic pain.

• Acute pain is mainly due to newly-onset mucosal lesions (cornea, oral cavity, oesophagus, anus or larynx and trachea).

• Chronic pain includes inflammatory, neuropathic and bone pain.

• The general conditions and nutritional status affect the severity of chronic pain.

• Evaluation of pain is mandatory and should address all the above mentioned components. It can be difficult in particular in children. Pain evaluation scales and frequency are the same used for non-EB patients and vary according to the patient age and on-going procedures.

• An EB expert should examine and listen attentively to the patient and his family in order to design the individual therapeutic strategies.

• An early adequate management of the physical pain is mandatory in order to reduce also the psychological pain component and to promote patient compliance.

• An early and regular psychological support for patients and families can contribute to the global efficacy of pain management.

• Unfortunately, in some cases, both pharmacological and psychological management strategies do not achieve complete pain control.

### General measures

#### Pain related to nursing procedures

•Bathing and dressing should be performed in a room equipped with all the necessary materials prepared in advance and close at hand (Figure 1). Non-pharmacological treatments based on cognitive-behavioural techniques are essential. A quiet and relaxing environment is helpful (music, films, pacifier dipped in glucose solution, etc.). The caregiver should provide contact, caresses and sweet words. Hypnosis can be useful in older children and adults.

• The choice of pharmacological treatment depends on the patient age, psychological status, type and severity of pain and planned procedure. The time of drug administration before the procedure varies according to the type of drug and route [68-73].

• Topical anaesthetics (xylocaine, lidocaine-prilocaine) are recommended before the care of painful tense blisters or wounds and venipuncture. However, the total dose should be limited to prevent the risk of seizures and methemoglobinemia.

• Paracetamol is the first choice for mild pain and short procedures. In more severe pain and complex procedures, opioids are indicated (from codeine to oxycodone and morphine). Opioid side effects include constipation, pruritus and rarely respiratory failure. The development of tolerance leads to dose increase with risk of addiction [70-74].

• Hydroxyzine and midazolam can be associated to analgesics to reduce anxiety and for short sedation. More rarely, ketamine is also used, particularly in children [75,76].

• Meopa® (nitrogen monoxide-oxygen mixture) can be helpful in most severe pain both in children and adults. Its use is restricted to the hospital setting and limited in time.

#### Chronic pain

• General measures are essential (e.g. music, yoga, relaxation techniques, hypnosis, etc.). In most cases, they need to be combined with analgesics. Paracetamol is usually the first-line treatment, followed by opioids (codeine, morphine, etc.). Non steroid anti-inflammatory drugs (NSAID) should be cautiously administered in patients with chronic/severe infections.

• A neuropathic pain component can be managed with pregabalin (Lyrica®) or gabapentin (Neurontin®) [73]. Tricyclic antidepressants, e.g. amitriptyline, are an alternative treatment.

• The daily analgesic dose should be distributed over 24 hours and increased before any care procedure.

• Bone pain, usually observed in severe RDEB, is due to abnormal bone mineralization, in turn related to poor nutrition, reduced mobility and chronic inflammation. Therefore, the improvement of nutritional status and physiotherapy contribute to reduce pain. Biphosphonates are useful to decrease bone pain, but their administration can be limited by perfusion difficulties and side effects (e.g. mandible osteonecrosis).

#### Acute pain

• Reassure the patient and his family.

• Administer adequate analgesic therapy (frequently opioids) or increase the dose of the ongoing treatment.

• Local anaesthesia may be useful in case of painful constipation due to anal erosions or fissures. Botulinum toxin for anal sphincter relaxation may represent a valid alternative.

#### Psychological pain

• Acute and chronic pain induce psychological pain since the first days of life.

• An inadequate pain control during procedures may cause a vicious circle with a memorization of the physical pain, and then anticipation and psychological distress increasing the physical pain at each procedure. Relaxation techniques, psychological support and pharmacological treatment should be provided.

• Psychological support should be reinforced during adolescence. A latent depression is not exceptional in the most severe forms and appropriate treatments have to be administered.

### Nutritional aspects

Growth and nutritional condition are major outcomes in EB patient care. Nutrition is often a real challenge. Blisters and erosions affecting oropharynx and oesophagus cause pain and dysphagia, followed by microstomia, ankyloglossia and tooth decay in severe RDEB. Altogether these manifestations contribute to reduce oral intake. Recurrent blistering can also lead to strictures and even complete oesophageal obstruction which are usually managed by endoscopic dilation [77-79].

On the other hand, protein-calorie and micronutrient needs are increased by (i) accelerated skin turn-over, (ii) blood and protein losses through skin wounds, (iii) recurrent infections, and (iv) chronic inflammation. Micronutrient deficiencies (iron, zinc, selenium, vitamins, etc.) can lead to severe complications. Iron, folate and vitamin B_12_ deficiencies contribute to multifactorial anemia typical of severe RDEB and JEB. Other nutrient deficiencies (carnitine, selenium) might play a role in inducing cardiomyopathy. In addition, an insufficient fluid and fibre intake frequently causes constipation, which can induce painful defecation (anal fissures). Overall, nutritional compromise is more important in generalized forms of RDEB and JEB [80-82].

Unfortunately, an improvement of nutrition in severe EB subtypes is usually not associated with a significant amelioration in wound healing rates. Nevertheless, an appropriate nutritional management is necessary since infancy also to promote pubertal development.

#### General principles

• The evaluation of patient growth, performed according to international standards (weight for length < 2 years; body mass index > 2 years), can be hampered by fixed contractures.

• The nutritional support should start early, especially in severe generalized subtypes.

• Dietetic advices, aimed to increase energy and protein content of oral food, should be addressed continuously to patients and their families.

• All factors affecting the quality of nutrition need careful management and follow-up: trauma related to hard food, dental caries and periodontal inflammation, gastro-oesophageal reflux, oesophageal strictures, inflammatory bowel disease, anal fissures and constipation, etc.

#### General measures

• Proteins and energy

Conventional dietetic approaches using oral high-energy and high-protein semi-liquid supplements rarely provide sustained improvement of dietary intake in severe EB forms. Consequently, children who survive without nutritional support become thin and short adults. A specific *T*ool to *H*elp *I*dentify *N*utritional *C*ompromise (*THINC*) in EB has been designed. The suggested nutritional requirements reach about 100-150% of the recommendations for healthy age- and gender-matched children for energy and 115-200% for proteins [81].

• *Micronutrient requirements*[81-87]

Evaluation of iron needs is frequently hampered by chronic inflammation. Daily intake of iron is recommended in case of hypochromic microcytic anemia; however it is often associated with gastric irritation, diarrhea or constipation. If oral supplementation is not tolerated and/or insufficient, intravenous administration (Venofer®, Ferinject®) should be considered and its frequency adapted to each patient. The use of erythropoietin or darbepoetin alfa in combination with iron supplementation has been proposed [85]. Vitamin requirements should be evaluated considering the disease type and patient age. In more severe forms, vitamin (A, C, D, E) levels should be checked at least once a year. Vitamin C supplementation is useful to enhance iron absorption. Vitamin D and calcium supplementation are needed to prevent/treat osteoporosis. Zinc, carnitine and selenium levels should be evaluated at least once a year in the most severe forms [88].

• *Nutritional procedures: nasogastric and gastrostomy feeding*

*Nasogatric feeding* can induce internal friction, irritation of nostrils and hypopharynx or oesophageal erosions. Therefore it should be used only for short periods in presence of severe oral cavity and pharyngeal blistering. The tube should be soft and of small size.

*Gastrostomy feeding (GTF)* has been shown to be well-tolerated and to improve patient nutritional outcome, growth and puberty. It should be performed in patients unable to feed orally (e.g. very severe and chronic oral lesions or oesophageal strictures not susceptible to dilation treatment) and/or presenting loss in weight and height of at least 1 standard deviation compared with their best growth level, despite regular nutritional advice. Starting GTF before malnutrition onset also reduces the problems linked to the choice of the button device and to the insertion site dehiscence [89-91]. A regular surveillance is necessary in order to prevent and/or early treat the possible local complications (chronic wound and infection).

The rhythm and rate of GTF delivery, volume, energy intake and formula concentration should be adapted to individual needs and tolerance and to patient and family lifestyle. The use of anti-secretory and anti-acid drugs (e.g. ranitidine or proton pump inhibitors) is recommended to treat gastro-oesophageal reflux and also to prevent skin damage due to gastric acid leakage at gastrostomy site [91]. GTF also easies the supply of micronutrients (iron, zinc, selenium, vitamins), nutritional supplements or drugs.

Oral feeding and swallowing skills should be continuously encouraged while using GTF. The patient should participate to “familial life” and familial meals. In all cases, a psychological support is essential. After the achievement of puberty development gastrostomy can be removed, provided a sufficient oral nutrition and absence of intractable oesophageal strictures. Unfortunately in JEB-Herlitz, GTF is not successful and thus not recommended in the context of palliative care [92].

### Physical therapy and rehabilitation

Physical and occupational therapy must be started early in life in particular in EBS with muscular dystrophy, generalized RDEB and JEB subtypes. The continuing work on muscles and joints delays contractures and deformities, improves functional mobility, enhances patient autonomy and, ultimately, promotes social inclusion. Static (preventive) and dynamic (corrective) orthosis, directed therapeutic exercises and recreational activities are powerful tools. Some forms of physical medicine, such as hydrotherapy, are also useful [93,94].

### Early diagnosis of squamous cell carcinoma (SCC)

SCC represents one of the major complications of EB, in particular generalized RDEB subtypes and, less frequently, generalized non-Herlitz JEB [5,12]. It occurs more often from the third-fourth decade of life. SCC usually arise in areas of chronic wounds and scars. The clinical presentation is frequently atypical with warty or ulcerated appearance. An early diagnosis is mandatory and literature data highlight that a diagnosis delay implies a poor prognosis.

In order to ensure an early diagnosis, it is necessary to perform [95]:

• whole-body physical examination, including the scalp, oral mucosa and genital area, every 6 months in RDEB and adult JEB patients;

• more frequent follow-up in presence of chronic hyperkeratotic or warty wounds;

• multiple excisional biopsies in case of crusted-warty skin lesions or chronic wounds unresponsive to proper treatments;

• early and wide excision of the lesion in case of histologically confirmed carcinoma.

### Management of intercurrent or associated cutaneous diseases

A few reports describe cutaneous infectious diseases occurring in EB patients, and even fewer data exist about associated chronic inflammatory diseases (psoriasis, atopic dermatitis, etc.) [96-99].

Skin diseases, when manifesting in EB patients, can:

• represent a diagnostic challenge due to atypical presentation (impetigo, scabies, etc.);

• worsen EB course, due to the development of pruritus and risk of infection (e.g. atopic dermatitis, scabies, VZV infection, impetigo, etc.);

• need an adapted management which should take into account the presence of EB lesions and the increased risk of percutaneous absorption. Some topical products may indeed cause irritation and toxicity (e.g. topical treatment for cutaneous parasitic infestations, such as benzyl benzoate, permethryne).

When dealing with these intercurrent/associated diseases, the principles for diagnosis and therapy are:

• Pruritus onset/worsening in EB patients should foster investigation for parasitic infestation such as scabies and lice.

• Pruritus in associated cutaneous diseases should be treated as described for pruritus in EB patients (Table 6) in order to prevent worsening of EB lesions.

• Ivermectin represents a valid alternative to standard topical treatment for parasitic infestations in EB patients with generalized disease [96,97].

### Therapeutic patient education

Therapeutic patient education, as defined by the World Health Organization (WHO), is a continuous process of patient-centered medical care, enabling patients affected by chronic diseases, and their families, to better manage their illness [100]. Patient education has been shown to also contribute to prevent complications and to improve quality of life (QoL).

Despite the lack of controlled studies, patient education is unanimously considered extremely important also for EB patients and their caregivers.

Table 8 summarizes the principles and measures of therapeutic patient education applied to EB.

**Table 8:** Patient education in epidermolysis bullosa: principles and contents

**Principles**	• Should be addressed to the patient, his/her family and caregivers
	• Should be delivered individually or in group
	• Should be adapted to the EB subtype, patient age, socio-cultural milieu and compliance
	• Should be delivered by specialized nurses with the support of the members of the multidisciplinary team and the psychologist
	• Should be performed orally and gradually, complemented by the release of information sheets for the patient and his/her family/caregivers
**Contents**	• Should at first clearly communicate the diagnosis, disease course and complications
	• Should give clear explanations on the need of an adapted genetic counselling
	• Should train the patient and his/her family/caregivers on the management of cutaneous and extracutaneous manifestations
	• Should educate the patient and his/her family/caregivers to early recognize infection signs and atypical aspects of the chronic wounds, and to consequently request a rapid evaluation by a dermatologist
	• Should provide training on the life style in order to prevent disease worsening

### Care of disease burden

EB has a major impact on the QoL of the patient and his family, starting since birth when the baby diversity is immediately perceived by the parents and the transmitted notion of skin fragility can hamper the development of normal affective relationships within the family. Isolation, fear or insecurity about infant care, breastfeeding, where to go for help, role adjustment, fatigue, relationship with siblings or partners, body image, nutrition, and the need for peer support - all must be dealt with [101-107].

Specific problems can then manifest at every age, they range from the patient perception of his diversity to disease limitations in daily life and challenges related to social integration. Coping with continuous pain and consciousness of disease worsening represent major challenges for these patients and their families, in particular in severe disease forms.

#### General principles

• Whenever possible, the transfer of the newborn to the centre of expertise should be performed when also the mother is able to move, avoiding disruption of the close parent-infant relationship or even the infant refusal.

• A gradual and multistep delivery of information on diagnosis and prognosis can reduce the psychological trauma. Particular caution should be taken to avoid culpability feelings in the parents.

• The involvement of both parents in the education process may facilitate their coping with the disease, reduce couple difficulties and reinforce their relationship.

• The parents should be accompanied in the educational process of the affected child, at the same time ensuring that the child progressively acquires as much autonomy as possible.

• The participation of the entire family can reduce the risk that non affected siblings feel neglected by the parents.

• A psychological support to the patient and his family is frequently needed and should be integrated in the multidisciplinary management process.

• Monitoring of the emotional status of the patient and/or his family and early diagnose psychological distress require yearly appointments with a psychologist even in absence of specific symptoms.

• Specific instruments should be used for evaluation of psychological status and QoL [108-110].

• The psychologist should be involved early when the newborn parents manifest signs of persisting distress.

• Indications to the provision of psychological and psychotherapeutic support to the patient and family members are summarized in Table 9.

**Table 9:** Indications to psychological or psychotherapeutic support to the family member(s) and patient

**Family members**	• Fear to breastfeed or handle the newborn and/or infant refusal
	• Lack of self-confidence or inadequacy feeling in coping with the disease
	• Anxiety to be left alone with the disease
	• Depression or disease refusal by one the two parents
	• Altered relationship of the couple (e.g. lack of interest in carrying out activities as a couple, loss of intimacy, negative impact on sexuality, etc.)
	• Culpability feelings and inability to take care of the non-affected children
	• Discomfort feelings or depression of the siblings
**Patient**	• Stress or depression related to the visibility of disease manifestations and the feeling of being different
	• Chronic pain exacerbated by daily care procedures
	• Chronic itching resistant to therapy
	• Stress or depression due to limitations in daily activities and social life
	• Lack of compliance and adherence to treatment, particularly in
	• Adolescence and adulthood

### Continuity of care

A well-organized and structured continuity of care is important in EB like in all chronic and rare diseases. Following hospital discharge, EB patients require social support and medical assistance for EB-related and non-related problems. The center of expertise stays as the main structure offering specialized care to EB patients and, at the same time, should ensure an adequate liaison with the community healthcare system. Public health and support services, both professional and peer, vary greatly country by country and even within the same country region by region. Nevertheless, some general rules can be drawn. At hospital discharge:

• a detailed referral form must be delivered and addressed to the primary care physician (pediatrician or general practitioner). It must report the diagnosis and the care given during hospitalization, describe the treatment plan for patient home care, the follow-up schedule and report the contact details of the EB team coordinator or his/her delegate from the center of expertise (e.g. specialized nurse) and, whenever available, of hotlines/call centres for emergency or urgent professional information;

• whenever foreseen by national laws, a certificate stating the diagnosis and a treatment plan must be addressed to the relevant public health authority in order to ensure free-of-charge care and provision of needed drugs, dressings and devices;

• depending on the national laws, ad hoc certificates must also be addressed to the public health service in order to guarantee homecare by specialized nurses, and if necessary also psychological support, physical therapy, occupational therapy, assistance by social workers, etc.;

• in countries where specialized EB nurses are not available, ad hoc training of the community health nurses is recommended;

• information sheets should be provided to the family/caregiver (relatives, friends, teachers, colleagues, etc.) in order to promote an adequate relationship with the patient, to explain the impact of the disease on daily activities and QoL (education, work, household chores, life management);

• information about the opportunity to get in touch with the patient association, if present in the country, should be released as detailed below.

### The relationship with the patient association

In most countries, DebRA is the EB patient association. All national DebRA belong to an umbrella organization known as DebRA International (http://www.debra-international.org).

• Patients should be informed about the presence of the patient association in their country and the interest to become a member.

• Patient associations contribute to improve patient access to information, reference centres, and social services.

• They also facilitate contact between patients and sharing experience regarding daily life.

• In some countries, they provide financial support and/or fund nurse home care and/or organize vacations for patients and families.

• Finally, they can contribute to promote professional training and research.

## Summaries

The optimization of EB patient healthcare requires the implementation of a wide range of measures. Towards this goal, the establishment in each country of expertise centres, which guarantee a multidisciplinary care, is essential. Equally important is the sharing of standards of care among expertise centres. The present recommendations should easy this process, support clinicians involved in EB care and, overall, contribute to improved patient care and QoL. Nevertheless, multicentre trials are needed, in particular to standardize interventions in wound care, itch and pain management, treatment of pseudosyndactyly and prevention and therapy of squamous cell carcinoma [38]. Finally, recent progress in the development of different gene, protein and cell therapy approaches open new perspectives for the treatment of these patients. In addition to the pilot study published by Mavilio and coworkers showing the feasibility of ex-vivo gene therapy in generalized non-Herlitz JEB [111], the results of clinical trials based on the use of (i) allogenic fibroblasts for local RDEB wound care [112,113] and (ii) allogenic hematopoietic stem cell/mesenchymal cell transplantation for severe RDEB forms [114] have been recently described. In particular hematopoietic stem cells transplantation has been reported to result in significant disease improvement, but not cure, in the majority of treated patients and further studies are ongoing to minimize the severe risks associated with transplantation procedure and to optimize the treatment protocol [114]. These results indicate that effective and specific EB treatments able to block disease progression are likely to become available in the medium term.

## Consent

Written informed consent was obtained from the participants for publication of this review and accompanying images, with additional parental written consent from those under 18 years of age.

## Abbreviations

EB: Epidermolysis bullosa; EBS: Epidermolysis bullosa simplex; DEB: Dystrophic epidermolysis bullosa; GTF: Gastrostomy feeding; JEB: Junctional epidermolysis bullosa; KS: Kindler syndrome; QoL: Quality of life; RDEB: Recessive DEB; SMARs: Silicone medical adhesive removers.

## Competing interests

MEH has received travel support from Mölnlycke and Pierre Fabre. GT has received travel support from Pierre Fabre. GZ, EBL, AC, CBu, SHR, AD, CFG, AHM, RDLL, MDV, GS, CDR, SLM, CBo have no competing interests.

## Authors’ contributions

MEH, GZ, CBo decided the topics to be developed and the methods to be employed, wrote the first recommendation draft and supervised the entire process of recommendation elaboration. EB and AC equally contributed to recommendation preparation: EB performed the literature review and contributed to «care of the EB newborn and infant», «EB care from childhood to adult», «management for surgical procedures», «pain management», «nutritional aspects»; AC performed the literature review and contributed to the «wound care», «skin care», «itch management», «continuity of care», and «management of intercurrent or associated diseases» topics. CBu contributed to «management for surgical procedures», «pain management» topics; SHR contributed to the «care of disease burden» topics, and revised the entire text; AD performed the literature review and contributed to the «wound care», «skin care» topics; GT contributed to «diagnosis of squamous cell carcinoma» and «therapeutic education» topics; CDR contributed to the «care of disease burden» topic; GS contributed to the part on care of newborn and infant; CFG contributed to the «skin care» and «wound care» topics; AHM and RDLL revised the entire text, and contributed to the «occupational therapy» topic and wrote the conclusions; MDV revised the entire text; SLM contributed to the literature review and revised the entire text. All authors have seen and approved the final manuscript.
